# Quadruple Hernia: A Rare Case Report

**DOI:** 10.7759/cureus.70962

**Published:** 2024-10-06

**Authors:** Pravin S Mane, Pushkar Galam, Dakshayani S Nirhale, Romi H Gaudani, Pragna Puvvada

**Affiliations:** 1 General Surgery, Dr. D. Y. Patil Medical College, Hospital and Research Centre, Dr. D. Y. Patil Vidyapeeth (Deemed to be University), Pune, IND

**Keywords:** bilateral, inguinal hernia, occult hernias, quadruple osteotomy, rare

## Abstract

Occult hernias, which are not detectable through clinical examination but can be identified during surgery, are typically asymptomatic. Bilateral inguinal hernias occur frequently, and in some cases, unilateral or bilateral inguinal hernias are observed in conjunction with paraumbilical hernias. However, it is rare to repair more than three hernias, including rare types, in a single procedure. Here, we describe a case of a 59-year-old male patient who underwent laparoscopic mesh repair for a right inguinal hernia, a left Spigelian hernia, an umbilical hernia, and an obturator hernia that was hidden and identified during surgical exploration. The report highlights the feasibility of repairing multiple abdominal hernias in a single setting, demonstrating the benefits of reducing the need for multiple operations, lowering costs, and decreasing the duration of hospital stays.

## Introduction

An abdominal hernia is characterized by the protrusion of a portion of the contents of the abdominal cavity through a weakened region of the abdominal wall [1]. Various factors, including weight lifting, obesity, prostatomegaly, chronic obstructive pulmonary disease, constipation, smoking, and increased intra-abdominal pressure, contribute to the development of abdominal hernias [2,3]. Herniation is more prevalent during pregnancy due to the laxity of pelvic ligaments caused by hormonal changes, and in the elderly due to degenerative muscle weakness. There is substantial evidence that multiple abdominal wall hernias may result from a collagen deficiency, characterized by increased type 3 collagen instead of type 1 collagen, and inadequate collagen cross-linking [4].

Unusual hernias, which are rare, include Spigelian hernias, obturator hernias, lumbar hernias, interparietal hernias, sciatic hernias, and perineal hernias. The diagnosis of abdominal hernias typically involves taking a thorough history of the patient's diet, lifestyle, and comorbidities, along with a physical examination [5]. Diagnostic tests are essential in challenging cases such as occult hernias [5,6]. For hernias not clinically visible, USG is the first-line radiological investigation [7]. However, MRI provides a better diagnostic picture than both CT scans and USG for hernia diagnosis [8].

## Case presentation

A 59-year-old male presented to the outpatient department with complaints of swelling in the right groin, left lumbar region, and umbilical region for six, four, and three years, respectively. The swellings were spontaneously reducible upon lying down and exhibited no signs of obstruction or strangulation, such as pain, nausea, vomiting, or skin changes. The patient had no addictions such as smoking, tobacco chewing, or alcohol consumption. Additionally, the patient had no comorbidities like obesity, steroid use, diabetes mellitus, chronic obstructive pulmonary disease (COPD), congestive heart failure (CCF), chronic liver disease (CLD), chronic renal failure (CRF) and had previously undergone a left-open inguinal meshplasty 10 years ago.

General physical examination findings were within normal limits. Abdominal examination revealed a 10 x 8 cm swelling in the right inguinoscrotal region, another 5 x 6 cm swelling in the left lumbar region, and a 2 x 1 cm swelling in the umbilical region. All swellings were soft, non-tender, and reducible, with a positive cough impulse.

**Figure 1:**
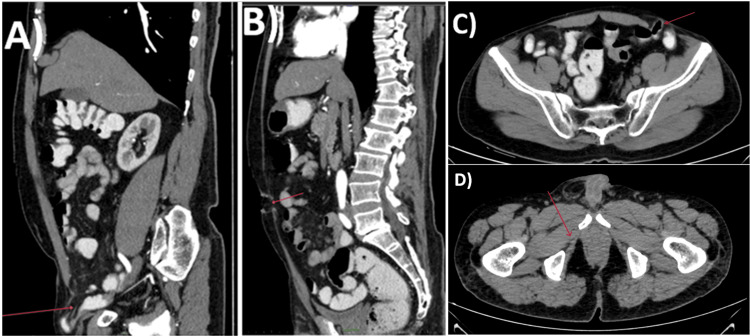
CT scan abdomen and pelvis. A) The sagittal section of the contrast-enhanced computed tomography (CECT) of the abdomen and pelvis shows a right inguinal hernia containing an ileum loop and omentum as its contents. B) Sagittal section of CECT of the abdomen and pelvis showing a defect measuring 12 x 10 mm (craniocaudal x transverse) in the anterior abdominal wall in the umbilical region within the linea alba, with herniating omentum as its content, suggestive of an umbilical hernia. C) Axial section of CECT of the abdomen and pelvis showing a defect measuring 52 x 35 mm (craniocaudal x transverse) in the anterior abdominal wall in the left iliac region at the lateral border of the rectus abdominis muscle, with herniating proximal sigmoid colon loop and mesentery as contents, suggestive of a left Spigelian hernia. D) Axial section of CECT of the abdomen and pelvis showing the right obturator foramen.

A CT scan of the abdominal and pelvic region revealed a defect measuring 52 x 35 mm (craniocaudal x transverse) in the left iliac region located on the anterior wall of the abdomen at the lateral border of the rectus abdominis muscle, containing herniating loops of the proximal sigmoid colon and mesentery, indicative of a left Spigelian hernia. A right inguinal hernia was noted, containing an ileum loop and omentum as its contents, likely direct, as seen medial to the inferior epigastric vessels. Additionally, a defect measuring 12 x 10 mm (craniocaudal x transverse) in the anterior abdominal wall in the umbilical region in the linea alba, with herniating omentum as its content, suggested an umbilical hernia (Figure 1).

All routine investigations were within normal limits. The patient did not have a collagen disorder. A decision was made to perform a laparoscopic repair of all three identified hernias, the right inguinal hernia, the left Spigelian hernia, and the umbilical hernia, during a single operative session. Ports were strategically positioned to address all three hernias concurrently. Intraoperatively, while visualizing all hernia sites through the camera port, an incidental finding of a left obturator hernia was discovered.

Consent was obtained for laparoscopic repair after informing the patient about potential complications of addressing multiple hernias in a single procedure. These risks included bleeding, seroma, hematoma, bowel injury, fistulation, chronic pain, recurrence, foreign body sensation, and mesh-related complications such as infection and erosion. The surgery was performed under general anesthesia, with epidural anesthesia provided for postoperative pain management. A 10mm camera port was placed below the left costal margin at Palmer’s point, allowing visualization of all hernia sites. During this process, an incidental left obturator hernia was identified.

**Figure 2:**
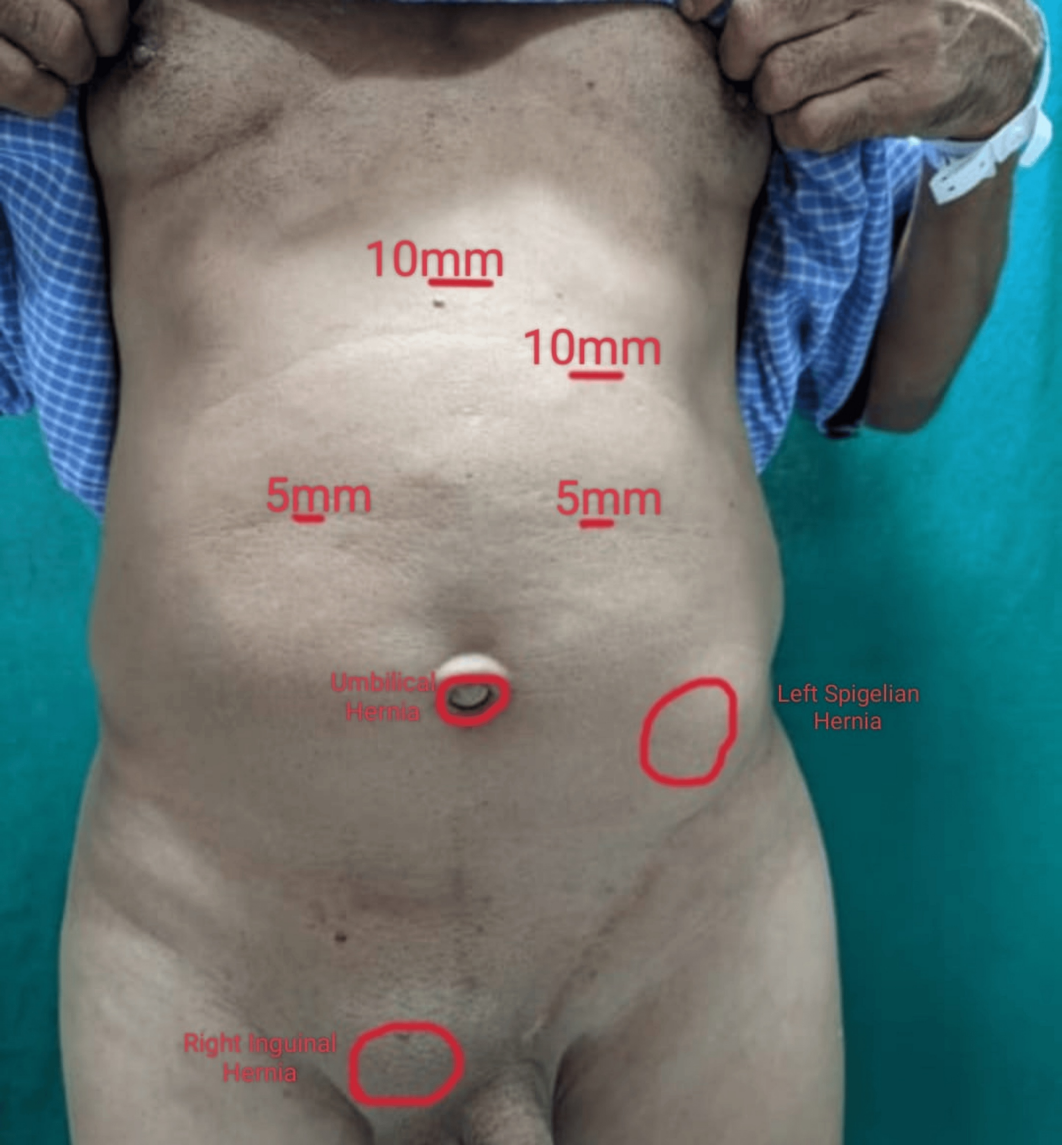
Port placement.

Additional ports were placed: a 10mm subxiphoid and two 5mm ports at the midclavicular line on either side, above the level of the umbilicus. The contents of all the hernias were reduced, and intraoperatively, the hernia sizes were measured as follows: a 5 x 3 cm Spigelian hernia, a 2 x 2 cm right inguinal hernia, a 1 x 1 cm umbilical hernia, and a 1 x 1 cm obturator hernia. An incision was made over the peritoneum to create space in the preperitoneal plane, where all the hernia sacs were dissected. The umbilical hernia defect was primarily repaired using Prolene (Figure 2).

A polypropylene mesh measuring 15 x 15 cm was used to cover the umbilical and left Spigelian hernias, while a polypropylene mesh measuring 12 x 15 cm was used to cover the right inguinal and right obturator hernias in the preperitoneal plane. The peritoneum was closed with continuous sutures. The use of polypropylene mesh made the procedure cost-effective.

Postoperatively, the patient remained stable without any complications and a pain score of 3. The patient was discharged on postoperative day 5 and followed up one week later for suture removal, with monthly follow-ups for six months. During this period, there was no evidence of surgical site infection or hernia recurrence.

## Discussion

Hernia is a prevalent medical issue, with an estimated 5% of the population developing this condition. Among these, approximately 75% are inguinal hernias, with two-thirds being indirect and one-third direct [9]. Indirect inguinal hernias are the most common, occurring in both men and women. Incisional and ventral hernias constitute 10% of all hernias, while femoral hernias make up 3% of groin hernias [10]. The incidence of groin hernias is 25 times higher in males compared to females. Femoral and umbilical hernias show a female predilection with ratios of 10:1 and 2:1, respectively [11].

Spigelian hernia, a rare ventral hernia, occurs through the Spigelian fascia, with the incidence ranging from 0.1% to 2% [12]. Obturator hernia, involving the protrusion of abdominal content through the obturator foramen, accounts for only 1% and is attributed to a higher morbidity and mortality rate (15-25%) due to delayed diagnosis and a high incidence of infarcted bowel (60-75%) [13]. Preoperative diagnosis is challenging, with most cases identified intraoperatively. Although the Howship-Romberg sign is characteristic of obturator hernia, it has only been reported in 15-50% of cases [14].

Not all abdominal wall hernias require repair. For asymptomatic patients with reducible hernias, a 'watchful waiting' approach is advisable. Hernia repair is necessary when patients present with signs of strangulation, such as pain, tenderness, and skin changes. Femoral hernias, carrying a higher risk of strangulation, should be repaired promptly upon diagnosis. Abdominal hernias can be managed with either open or laparoscopic mesh repair; however, laparoscopic mesh repair is the preferred method for recurrent and multiple hernias. Multiple hernia repairs in a single patient setting are rare [15].

Goldberger HA et al. reported a case involving multiple hernias, including an umbilical hernia, epigastric hernia, ventral hernia, and a spigelian hernial sac containing a mesocolic hernia. In this case, the four hernial defects were converted into a single defect, and the Mayo principle of repair was applied [16].

## Conclusions

The preoperative diagnosis of obturator hernia is particularly challenging, making it essential for surgeons to have comprehensive knowledge of all potential hernia sites and to thoroughly examine them during surgery. Repairing four hernias, including rare types, can be effectively accomplished laparoscopically in a single setting. Laparoscopic hernia repair is the preferred procedure for addressing multiple hernias simultaneously. However, expertise in laparoscopic techniques is essential for successful outcomes. Polypropylene mesh should be positioned in the preperitoneal plane to minimize the risk of mesh-related and bowel-related complications. Utilizing sutures for mesh fixation, instead of tackers, further reduces the cost of the procedure. This approach offers several advantages, including lower procedural costs, shorter hospital stays, and fewer postoperative complications, making it a highly effective and efficient option for hernia repair.
